# Oral health knowledge, attitude and practices of Bangladeshi female sex workers toward their 7-to-17-years old children: a cross-sectional study

**DOI:** 10.1186/s12903-025-05535-z

**Published:** 2025-02-13

**Authors:** Afia Mahmuda Khan, Sumaiya Zabin Eusufzai, Taseef Hasan Farook, Mehnaj Sharmin, Sabrin Shohid, Lameea Shahed, Tabassum Zerin, Diptendu Kumar Chanda, Sheikh Jamal Hossain, Mohammad Delwer Hossain Hawlader

**Affiliations:** 1 Department of Public Health, North South University, Dhaka, 1229 Bangladesh; 2 Public Health Promotion and Development Society (PPDS), Dhaka, 1205 Bangladesh; 3 Maternal and Child Health Division (MCHD), International Centre for Diarrheal Disease Research, Bangladesh, (icddr,b), Dhaka, 1212 Bangladesh; 4 School of Dental Sciences, University Sains Malaysia, 16150 Kelantan, Malaysia; 5 Adelaide Dental School, The University of Adelaide, Adelaide, SA 5000 Australia; 6 Department of Public Health and Hospital Administration, National Institute of Preventive and Social Medicine (NIPSOM), Dhaka, 1212 Bangladesh; 7 Global Health and Migration Unit, Department of Women’s and Children’s Health, Uppsala University, Uppsala, Sweden; 8 NSU Global Health Institute (NGHI), North South University, Dhaka 1229, Bangladesh

**Keywords:** Knowledge, Attitude, Practice, Oral health, Sex worker

## Abstract

**Background:**

Children born into prostitution often face significant barriers in accessing healthcare, including oral health services. This study aimed to assess the knowledge, attitudes, and practices (KAP) of female sex workers (FSWs) regarding their school-going children’s oral health, as well as the oral health status of these children in Dhaka, Bangladesh.

**Materials and methods:**

A cross-sectional study was conducted from March 2023 to February 2024 with a sample of 180 FSW mothers/institutional caregivers and their school-going children between ages 7 to 17. A semi-structured questionnaire was used to collect data on KAP. The children’s oral health was assessed using the DMFT/dmft index for caries and the gingival index (GI) for gingival health, while plaque and calculus levels were measured using the plaque index (PI) and calculus index (CI), respectively.

**Results:**

Among the FSW mothers/ institutional caregivers, 79% had good knowledge of oral health, 77.2% displayed a positive attitude, and 62.8% were informed about proper oral health practices. FSW mothers/ institutional caregivers who had higher educational attainment were three times more likely to practice good oral hygiene compared to those with lower-educated caregivers (OR = 3.27, β = 1.11, *p* < 0.05, CI = 1.36–7.87). Similarly, children whose mothers/caregivers had better oral health knowledge showed three times higher oral health practice scores compared to those with lower knowledge (OR = 3.20, β = 1.16, *p* < 0.05, CI = 1.36–7.87).

**Conclusion:**

The study suggests that while most FSW mothers and institutional caregivers possess adequate knowledge and a positive attitude towards their children’s oral health, many children, particularly those living with FSW mothers, continue to exhibit poor oral health practices. Higher educational attainment and better oral health knowledge among mothers/caregivers were key determinants of improved oral health practices in children.

**Supplementary Information:**

The online version contains supplementary material available at 10.1186/s12903-025-05535-z.

## Background

The term “sex work” replaces derogatory terms like “prostitution,” which historically implied immorality and illegality [1]. Sex workers engage in consensual sexual services or seductive acts in exchange for money or goods [2]. People enter sex work for various reasons, including choice, circumstances, or coercion, much like other forms of labor under capitalism [3]. In Bangladesh, there are approximately 200,000 female sex workers (FSWs) [4], dispersed across both urban and rural areas, working independently or within brothels [5]. Notably, Bangladesh is home to one of the largest brothels in the world, located in Daulatdia, Rajbari district, which accommodates about 2,000 sex workers [6, 7].

Over the past decade, the socioeconomic status of sex workers in Bangladesh has improved due to the efforts of NGOs and the legalisation of brothels and red-light districts. However, despite these advances, sex workers still face significant marginalization [8]. Social exclusion and stigma are critical factors shaping the economic realities of sex work [9, 10]. Mondal et al. reported that 96% of sex workers in Bangladesh are mothers, with an average of two children [11]. Due to the taboo surrounding prostitution, these children are often neglected, making them vulnerable and marginalized, which negatively impacts their physical and psychological development [12, 13].

Children from brothels are often perceived by society as the most “tainted” or “rotten.” Whether due to their direct involvement in the sex industry or simply by association, they carry a deep sense of shame about their origins [14]. These children grow up in a damaging social environment marked by alcoholism, substance abuse, limited access to healthcare and education, parental separation, and inadequate supervision. These circumstances often lead them to follow the same professions they were exposed to during childhood [15–17].

Oral disorders in these children are common and encompass issues such as dental caries, diseases of the oral mucosa, and periodontal disease. Dental caries, caused by the gradual decalcification of tooth enamel, is one of the leading oral health problems among children and is closely linked to poor oral hygiene practices [18–20]. The lack of access to proper dental care in such environments exacerbates these conditions, further impacting their overall well-being.

Oral diseases pose a significant burden on socially marginalized communities, largely due to barriers in accessing preventive care and treatment [21]. In rural regions of Bangladesh, particularly those with limited income and education, oral health services are underutilized because of restricted availability and access [22]. Poverty can lead to various issues, including financial instability, low maternal education, and insufficient knowledge, attitudes, and practices regarding children’s oral health. These challenges often prevent mothers from ensuring proper oral care for their children [23–25]. The critical role of mothers in shaping their children’s oral health behaviors has been well-documented [26, 27].

Although children of sex workers are a socially excluded group, most research has focused on their education, health, and social well-being, with little attention given to their oral health [11, 28]. Only one study has examined the oral health of female sex workers (FSWs), finding that many experienced dental caries, and those with HIV were more prone to periodontitis [29]. However, the oral health of their children remains underexplored. To address this gap, our research aimed to evaluate the knowledge, attitudes, and practices (KAP) of FSWs regarding their children’s oral health, as well as the oral health status of their children in Dhaka, Bangladesh.

## Materials and methods

### Study design

A cross-sectional study design was applied to assess the oral health knowledge, attitude, and practices of female sex workers and institutional caregivers regarding their children from March 2023 to February 2024. A convenience sampling technique was employed as it was very challenging to source the current sample due to the dearth of documentation available. Participants included FSWs, institutional caregivers, and their children aged 7 to 17 years old who attended dental camps in Daulatdia brothel and three shelter homes in Dhaka city. To maximize recruitment, participation was open to all attendees of the dental camps. However, many FSWs, institutional caregivers, and their children were not available in the centers for various reasons. Consequently, samples were selected on a first-come, first-enrolled basis.

### Study location and relevance

To gain access to the study area, we partnered with Project Pothchola, a program by the Give Bangladesh (GB) Foundation dedicated to supporting the children of sex workers. With their cooperation, the investigators were granted access to Daulatdia Brothel and three shelter homes in Dhaka city. For data collection, ‘Shishuder Jonno Amra’ (SJA), KK Foundation (KKF), two not-for-profit organizations, ‘BASHA’, a day care center in Dhaka city were selected. Additionally, ‘Mukti Mohila Shamity’ (MMS), an NGO near Daulatdia Brothel, was also selected. These centers were chosen based on their availability and alignment with the study’s inclusion criteria.

For context, BASHA rescues sex workers and their children from various brothels in Bangladesh, providing income-generation opportunities to sex worker mothers. Similarly, Shishuder Jonno Amra and KK Foundation offer residential, educational, and medical care to the children of sex workers. Mukti Mohila Shamity, established under the ‘Alternative Livelihood Opportunity’ initiative with funding from the ‘Foundation for Humanity and Global Affairs’ in Canada, focuses on child development programs for children from Daulatdia Brothel.

### Study sample

The sample size calculation was based on maternal oral health knowledge concerning their children in the context of Bangladesh. We used a standard error of 5%, a 95% confidence interval (CI), and a prevalence of 88.5% for mothers’ knowledge about their 5 to 9-year-old children in Dhaka city [30]. Our initial sample size calculation indicated 156 participants. To account for a 20% nonresponse rate, we adjusted the sample size to 188 participants. Data was collected from the four study locations using a convenient sampling method, with 50 samples from MMS Center, 66 samples from SJA, 9 samples from KKF, and 63 samples from BASHA. The study included mothers who were sex workers or institutional caregivers, along with their children aged 7 to 17 years who had no physical or mental health issues. According to UNICEF, primary education is designed for children aged 76 to 11, while high school education is for those aged 12 to 17 [31]. Due to accessibility barriers, we kept the sample size small. After adjusting for missing values and conducting data screening, the final sample size was 180 participants. Details of the sample size calculation are provided in ‘Additional File 2’.

### Ethics and informed consent

The study was conducted in accordance with the Declaration of Helsinki. Ethical clearance was obtained from the Institutional Review Board/Ethics Review Committee (IRB/ERC) of North South University, Dhaka, Bangladesh (2023/OR-NSU/IRB/0204). Prior to each interview, participants were thoroughly informed about the study’s objectives and methods.

Written consent was obtained from the mothers before the oral examination and data collection. Informed consent forms detailing the study’s objectives and methods were sent to the mothers a day before data collection. Literate mothers signed the forms, while those who were not literate had the forms read to them by someone who could read and write before signing.

For children aged 7–11 years, verbal consent was obtained, while individuals aged 12–17 years provided written informed consent in addition to their mothers’ written consent. For mothers who could not participate, data were collected from the caregivers of the children. Prior to the survey, consent forms were signed by the mothers or caregivers. Only female sex workers or caregivers and their children, who had provided consent, were included in the study.

### Data collection

Data collection involved administering a questionnaire on sociodemographic characteristics and oral health knowledge, attitudes, and practices through face-to-face interviews with each FSW mother or caregiver. Data was collected from caregivers of children residing in the two shelter home organisations and from FSW mothers of children living in the Daulatdia Brothel and one daycare center (BASHA) in Dhaka.

Oral clinical examinations of the children were conducted by four dentists. Two of these dentists were calibrated according to the standard procedures outlined by the World Health Organization. The examinations took place at the respective organizations or daycare centers, where the children were seated in chairs, and the examiner was positioned in front of them. Teeth were dried with cotton pellets, and both torchlight and natural daylight were used for better visibility. Caries were scored using the World Health Organization’s recommended methods, employing mouth mirrors and CPI ball-ended probes as examination tools [32].

### Research tool

A semi-structured questionnaire was used to assess sociodemographic information and the knowledge, attitudes, and oral health practices of mothers or caregivers regarding their children.

- Part A of the questionnaire gathered sociodemographic information, including the child’s age, sex, education, the mother’s or caregiver’s age, education, and whether the child resided with the mother or caregiver.
- Part B consisted of 12 questions assessing oral health knowledge.
- Part C included 9 questions on attitudes toward the child’s oral health, and.
- Part D contained 8 questions evaluating the child’s oral health practices.

The knowledge section covered topics such as causes of dental decay, prevention of oral health problems, brushing habits, the importance of dental visits, and the role of sugary foods and drinks in dental decay. The attitude section addressed the mother’s approach to preventing dental decay and maintaining dental hygiene. The practice section evaluated brushing teeth, cleaning the tongue, and overall mouth hygiene.

The survey questionnaire was adapted and modified from a validated KAP (Knowledge, Attitudes, and Practices) questionnaire used in a study by Sharmila et al. [33]. An expert panel comprising 5 professionals (2 from public health, 1 from social sciences, and 2 from dentistry) reviewed and provided feedback on the adapted questionnaire, which was then revised accordingly. The questionnaire was translated into Bangla from English, then re-translated into English, and subsequently translated back into Bangla. This forward and backward translation process was carried out by two language experts fluent in both Bangla and English.

A pilot study involving 50 mothers with school-going children was conducted to pretest the translated KAP questionnaire. Internal consistency among the questionnaire items was assessed using Cronbach’s alpha, with data from the pilot study. A Cronbach’s alpha score above 0.7 indicates high internal consistency, while scores between 0.40 and 0.69 suggest moderate consistency. Higher Cronbach’s alpha values reflect greater reliability of the questionnaire items [34, 35]. The Cronbach’s alpha values for the knowledge, attitude, and practice subscales were 0.81, 0.84, and 0.71, respectively, indicating good consistency for knowledge and attitude and acceptable consistency for practice items. The questionnaire is provided in the supplementary file.

For the knowledge section, responses were scored as 0 for “no” or “don’t know” and 1 for “yes.” In the attitude section, responses were scored as 0 for “disagree” and 1 for “agree.” In the practice section, scores ranged from 0 to 1 for some questions and from 0 to 4 for others. Scores for each section were summed and categorized based on Bloom’s cut-off points (where 80% is considered good and < 80% is poor for knowledge, positive and negative for attitudes, and good and bad for practices). This scoring technique is consistent with the methodologies of Bloom et al., Engelhart et al., and Furst et al. [36]. Oral health status of the children was assessed in organized dental camps in the four study areas. Prior to the initiation of these dental camps, six examiners were trained and calibrated for oral health status assessment. Intra and inter-examiner reliability tests were conducted to evaluate the consistency of dental caries (DMFT/dmft). Cohen’s Kappa statistic was used to assess the intra and inter-examiner reliability and was found to be 0.84 and 0.86, respectively.

### Assessment of oral health status

Following the World Health Organization (WHO) 2013 guidelines, dental caries was evaluated to determine decayed, missing, and filled teeth (DMFT) for permanent tooth indices and decayed, missing, and filled teeth (dmft) for deciduous teeth [37]. The total values of dmft and DMFT were calculated separately and jointly as the sum of d + m + f + D + M + F to obtain the numerical expression of caries prevalence. The scores were assigned, and the degree of severity of dental caries was defined as dmft + DMFT = 0 (caries-free) or dmft + DMFT > 0 (caries present) [38]. The individuals’ gingival health status was evaluated using the gingival index (GI) which was proposed by Silness and Löe in 1963 [39], and dental plaque was recorded in the sample under study using the Silness and Löe plaque index, modified by Löe in 1967 [40]. The calculus index method of Ramfjord (1959) was used to score mineralized deposits [41]. Disposable plastic mirrors, blunt edge caries probes, artificial light, and plastic chairs were used in data collection for DMFT. Two calibrated dentists determined the caries prevalence.

### Statistical analysis

Statistical analyses were conducted using IBM SPSS version 26. A frequency distribution test was performed to explain the variables. A chi-square test was employed to determine the associations between sociodemographic variables and oral health status variables with knowledge, attitude, and practices scores, with a statistically significant *p*-value of < 0.05. Logistic regression analysis was applied to investigate the associations between the education level of mothers or caregivers, whether children were living with FSW mothers or institutional caregivers, their knowledge, and their attitude toward the oral health practices of their children. The covariates of this model were selected based on variables having biological and scientific plausibility with the main outcome.

## Results

**Fig. 1:**
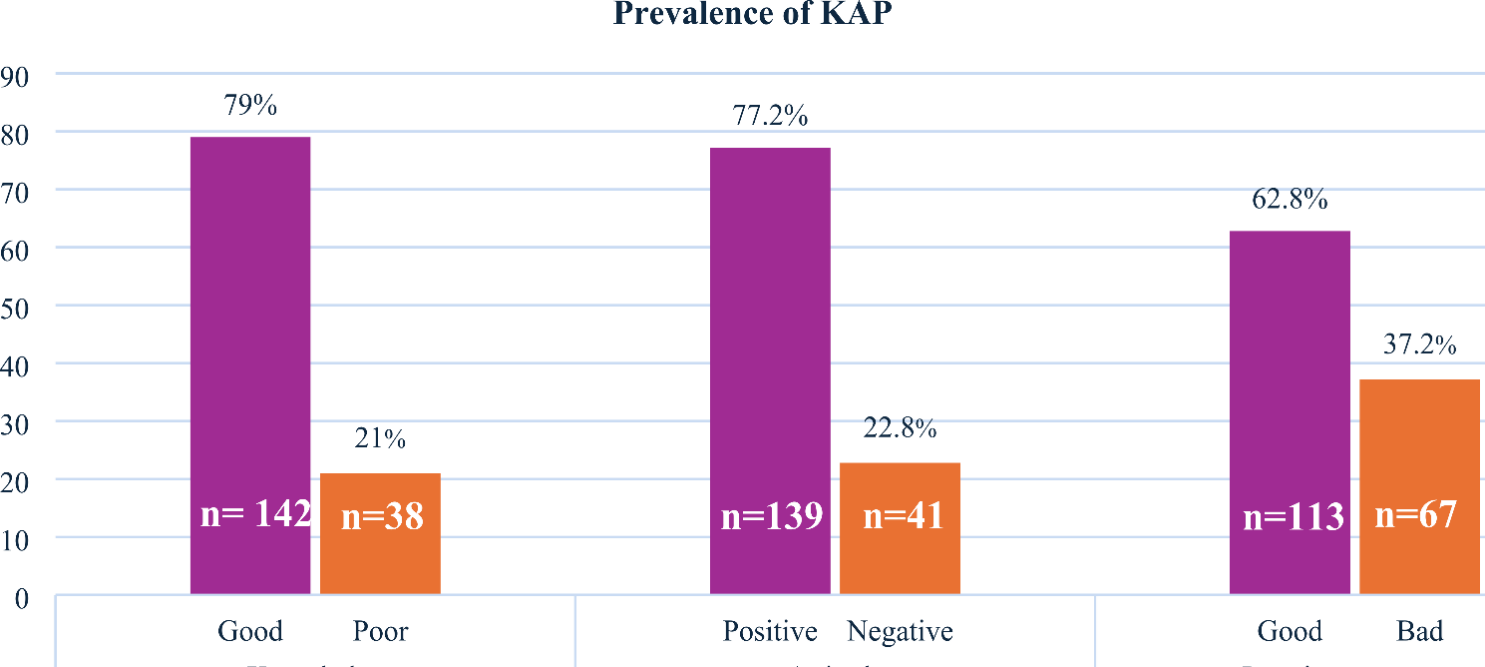
Prevalence of knowledge, attitude, and practices of FSW mothers/institutional caregivers

The mother’s/ institutional caregiver’s responses to knowledge, attitudes, and practices toward the oral health of their school children are provided in the supplementary Tables S1, S2 and S3, which are located in the supplementary file. The prevalence of KAP in sex worker mothers/institutional caregivers is displayed in Fig. 1. Greater than three-quarters (79%) of the mothers/caregivers had favorable knowledge regarding their children’s oral health. However, two-fifths (37.2%) of the surveyed mothers/caregivers acknowledged that their children had embraced bad oral health practices.

Table 1 reveals that out of the 180 participants, a significant proportion (58.9%) were female. The age distribution of the children showed that approximately 56.7% fell into the age range of 7–11 years. A majority of the FSW’s children (72.2%) resided in three different shelter homes in Dhaka city. In contrast, more than a quarter (27.8%) of the children were in the Daulaidia brothel. Around 41.1% of mothers/institutional caregivers were aged between 18 and 30 years. In addition to that, around 66.7% of FSW mothers completed primary education, while the figure for secondary or higher education was around 33.3%. More than half of the respondents (54%) were FSW mothers, whereas 46% of respondents o were institutional caregivers.

Regarding the oral health status-related information, over half of the surveyed children (52.8%) had caries, indicating poor oral hygiene. A majority of children (71%) had visible plaque, suggesting that most individuals had ineffective plaque control. Approximately 62.8% of children had signs of calculus. Conversely, it is notable that 65% of the children in the study displayed no signs of gingivitis, indicating a relatively favorable state of oral health in this group.

**Table 1:** Socio-demographic characteristics and oral health status of the respondents

Variables	Socio-Demographic Information	
	*N*	%
**Sex of children**		
Female	106	58.9
Male	74	41.1
**Age of children**		
7–11 years	102	56.7
12–17 years	78	43.3
**Age of mothers/caregivers**		
18–30 years	74	41.1
31–40 years	59	32.8
>=41 years	47	26.1
**Education of children**		
Primary School	102	56.7
High School	78	43.3
**Education of mothers/caregivers**		
Primary or less	120	66.7
Secondary or higher	60	33.3
**Type of caregivers**		
FSW Mother	97	54
Institutional Caregiver	83	46
**Place of residence**		
Daulatdia	50	27.8
Dhaka	130	72.2
**Caries**		
Caries Free	85	47.2
Caries Present	95	52.8
**Plaque**		
Absent	52	29
Present	128	71
**Calculus**		
Absent	67	37.2
Present	113	62.8
**Gingivitis**		
Absent	117	65
Present	63	35

The association of KAP of mothers/caregivers with sociodemographic details is illustrated in Table 2. Mothers or institutional caregivers aged between 31 and 40 years old and residing in Dhaka, were found to have significantly good knowledge (*p* < 0.002), a positive attitude (*p* < 0.004), and good practice (*p* < 0.032). The association between types of caregivers and KAP was found to be highly significant. It was observed that FSW mothers exhibited poorer knowledge (*p* < 0.001), more negative attitude (*p* < 0.001), and less favorable practice (*p* < 0.001) in relation to the oral health of their children, in comparison to institutional caregivers. Additionally, it was established that the mother’s/institutional caregiver’s practice toward their children was significantly associated with the education level (*p* < 0.002) and age category of the children (*p* < 0.002). However, there was no significant association found between the KAP of mothers/institutional caregivers with the sex of children and the educational level of mothers/institutional caregivers.

**Table 2:** Association between mothers’ knowledge, attitude, and practices and socio-demographic characteristics

Variables	Knowledge			Attitude			Practices		
Demographic Information	Good *n*(%)	Poor *n*(%)	*p*-value	Positive *n*(%)	Negative *n*(%)	*p*-value	Good *n*(%)	Bad *n*(%)	*p*-value
**Sex of children**									
Female	84(79.2)	22(28.2)	0.888	85(80.2)	21(19.8)	0.256	72(67.9)	34(32.1)	0.087
Male	58(78.4)	16(21.6)		54(73.0)	20(27.0)		41(55.4)	33(44.6)	
**Age of children**									
7–11 years	82(80.4)	20(19.6)	0.572	84(82.4)	18(17.6)	0.061	54(52.9)	48(47.1)	**0.002**
12–17 years	60(76.9)	18(23.1)		55(70.5)	23(29.5)		59(75.6)	19(24.4)	
**Age of mothers/caregivers**									
18–30 years	49(66.2)	25(33.8)	**0.002**	50(67.6)	24(32.4)	**0.004**	42(56.8)	32(43.2)	**0.032**
31–40 years	53(89.8)	6(10.2)		54(91.5)	5(8.5)		45(76.3)	14(23.7)	
>=41 years	40(85.1)	7(14.9)		35(74.5)	12(25.5)		26(55.3)	21(44.7)	
**Education of children**									
Primary School	82(80.4)	20(19.6)	0.572	84(82.4)	18(17.6)	0.061	54(52.9)	48(47.1)	**0.002**
High School	60(76.9)	18(23.1)		55(70.5)	23(29.5)		59(75.6)	19(24.4)	
**Education of mothers/caregivers**									
Primary or less	93(77.5)	27(22.5)	0.518	93(77.5)	27(22.5)	0.9	70(58.3)	50(41.7)	0.081
Secondary or higher	49(81.7)	11(18.3)		46(76.7)	14(23.3)		43(71.7)	17(28.3)	
**Types of caregivers**									
FSW Mother	63(64.9)	34(35.1)	**< 0.001**	61(62.9)	36(37.1)	**< 0.001**	47(48.5)	50(51.5)	**< 0.001**
Institutional Caregiver	79(95.2)	4(4.8)		78(94.0)	5(6.0)		66(79.5)	17(20.5)	
**Place of residence**									
Daulatdia	34(68.0)	16(32.0)	**0.026**	29(58.0)	21(42.0)	**< 0.001**	24(48.0)	26(52.0)	**0.011**
Dhaka	108(83.1)	22(16.9)		110(84.6)	20(15.4)		89(68.5)	41(31.5)	

Pearson’s Chi-square test, bold number indicate *p* < 0.05

The study showed a significant association (Table 3) between children who had caries-free oral health and their mother’s knowledge (*p* < 0.03) and practice (*p* < 0.003). 86% of the children’s caries-free status could be attributed to their mother’s good knowledge, while 74% could be attributed to their mother’s good practices.

**Table 3:** Association between mothers’ knowledge, attitude, and practices and their children’s oral health status

Variables	Knowledge			Attitude			Practices		
Oral Health Status	Good *n*(%)	Poor *n*(%)	*p*-value	Positive *n*(%)	Negative *n*(%)	*p*-value	Good *n*(%)	Bad *n*(%)	*p*-value
**Caries**									
Caries Free	73(86)	12(14)	**0.03**	68(80.0)	17(20.0)	0.401	63(74)	22(26)	**0.003**
Caries Present	69(72.6)	26(27.4)		71(74.7)	24(25.3)		50(52.6)	45(47.4)	
**Plaque**									
Absent	39(75.0)	13(25.0)	0.415	36(69.2)	16(30.8)	0.103	28(53.8)	24(46.2)	0.114
Present	103(80.5)	25(19.5)		103(80.5)	25(19.5)		85(66.4)	43(33.6)	
**Calculus**									
Absent	51(76.1)	16(23.9)	0.483	47(70.1)	20(29.9)	0.081	34(50.7)	33(49.3)	**0.01**
Present	91(80.5)	22(19.5)		92(81.4)	21(18.6)		79(69.9)	34(30.1)	
**Gingivitis**									
Absent	96(82.1)	21(17.9)	0.153	93(79.5)	24(20.5)	0.323	72(61.5)	45(38.5)	0.639
Present	46(73.0)	17(27.0)		46(73.0)	17(27.0)		41(65.1)	22(34.9)	

Pearson’s Chi-square test, bold number indicate *p* < 0.05

Table 4 shows predictors of having good oral health practices of children according to mothers or caregivers. Compared to mothers and caregivers aged 18 to 30 years, those aged 31 to 40 years were 2.45 times more likely to be aware of their children’s oral health practices (OR = 2.45, *β*:0.896, *p*-value = 0.02, CI = 1.15–5.24). Children of mothers/institutional caregivers with higher educational attainment were three times more likely to practice good oral health hygiene than the children of those with mothers/caregivers of low educational level (OR = 3.27, *β*:1.11, *p*-value = 0.008, CI = 1.36–7.87). Children who were living with mothers exhibited 87.4% less good oral health practices than the children who were living with institutional caregivers (OR = 0.126, *β*: -2.075, *p-*value = 0.025, 95% CI: 0.021–0.767). Similarly, children living with mothers/caregivers who had good oral health knowledge scores tend to have 3 times higher oral health practice scores compared to those with poor oral health knowledge (OR = 3.20, *β*:1.16, *p*-value = 0.049, CI = 1.36–7.87). However, no associations were found between oral health practice with attitude, place of residence and caries.

**Table 4:** Logistic regression model exploring the association of predictor variables with children’s oral health practices

Variables	OR	95%CI		*p*-value
Age of mothers/caregivers		Lower	Higher	
18–30 years	Reference			
31–40 years	2.449	1.150	5.215	**0.020**
>=41 years	0.943	0.452	1.97	0.877
**Education of mothers**				
Primary or less	Reference			
Secondary or higher	3.270	1.360	7.870	**0.008**
**Types of caregiver**				
Institutional Caregiver	Reference			
FSW Mother	0.126	0.021	0.767	**0.025**
**Place of residence**				
Dhaka	Reference			
Daulatdia	0.924	0.408	2.094	0.850
**Caries**				
Absent	Reference			
Present	0.570	0.287	1.131	0.108
**Knowledge**				
Good	Reference			
Poor	3.200	1.000	10.25	**0.049**
**Attitude**				
Positive	Reference			
Negative	0.490	0.120	2.000	0.320

Logistic regression analysis, bold characters indicate *p* < 0.05, OR: Odds ratio, CI: Confidence interval. Hosmer-Lemeshow test showed that *p*-value = 0.170 indicates that the model is a good fit for the data

## Discussion

The present study investigated the knowledge, attitudes, and practices of sex worker mothers regarding the oral health of their school-going children, an area not previously explored. This is the first study to specifically examine the oral health knowledge, attitudes, and practices of sex worker mothers concerning their children.

Despite the relatively high level of dental health knowledge among female sex workers and caregivers, this knowledge has not always been effectively translated into oral health practices for their children. Existing literature indicates that parental knowledge, attitudes, and beliefs play a significant role in influencing a child’s dental health [42]. Achieving good oral health condition by reducing caries prevalence is crucial for ensuring better quality of life among children [43]. In that regard, the findings revealed that these mothers/caregivers have a good level of knowledge (78.9%) about their children’s oral health. This level of knowledge aligns with previous studies conducted in populations with similar cultural norms [37]. However, findings from other populations showed contrasting results [44], which may be attributed to varying cultural and contextual factors across different countries. Mothers or careivers aged between 31 and 40 years had significantly better awareness of the practice of children’s oral health than mothers from age group of 18 to 30 years. This association was not consistent with other studies [45, 46]. However, within the over-41 age group, there was a decrease in oral health practices compared to the 18–30 group, though this difference was not statistically significant. The discrepancy may be attributed to the observation that mothers in the 31–40 age group tend to exhibit higher levels of education and greater attentiveness to their children’s oral health practices. Among those mothers who were well-informed about their children’s oral health practices, 52.6% had caries and 69.9% had dental calculus. This discrepancy highlights that knowledge alone does not necessarily ensure effective oral health practices.

Female sex workers face unique challenges, including stigma associated with their profession, HIV status, and human rights violations [47]. These factors add to their lower material standards of living measured by money, social class, and social support. The unfortunate combination has been shown to adversely affect their children’s oral health [48, 49]. This deterioration in oral health is evident regardless of whether clinical or self-reported indicators are used.

Children of female sex workers (FSWs) living in Dhaka shelter homes generally exhibit better oral health practices compared to those living in brothels. The demanding nature of sex work often leaves mothers with little time for their children, who may be perceived as “unwanted” [14].

The current findings also indicate that children of FSW mothers or institutional caregivers with higher educational levels demonstrate better oral health practices compared to those with less formal education. This suggests that maternal education significantly influences children’s oral health behaviours. Similar conclusions have been supported by studies conducted by Jain et al. and Vann et al. [50, 51]. Children living with female sex worker (FSW) mothers exhibit poorer oral health practices compared to those living with institutional caregivers. In Bangladesh, stigma and prejudice against sex workers often affect access to health care for their children. Female sex workers have reported experiencing humiliation and receiving less attention for both themselves and their children. Stigma, criminality, and other marginalizing factors hinder female sex workers from fully participating in motherhood and jeopardize the health, safety, and well-being of both themselves and their children [52].

Not-for-profit organisations, like BASHA, offer a sustainable livelihood to women escaping the sex trade, who face profound shame and a strong desire for freedom. The initiative provides literacy courses, vocational training, counselling, health services, and parenting classes. Additionally, BASHA’s daycare facility offers educational lessons and extracurricular activities for the children of these women while they are engaged in the program [53, 54]. This comprehensive support may help explain why children in institutional care settings show better oral health compared to those living in brothels.

The study identified several limitations. The sample was not fully representative of all sex workers and their school-going children in Bangladesh, as data were collected only from Dhaka city and Daulatdia. Access to participants from other parts of the country was challenging. Additionally, inquiries about the timing of the initial dental appointment and the onset of brushing and flossing could introduce recall bias. There is also a potential for social desirability bias, as institutional caregivers might exaggerate the oral health practices of the children to highlight the quality of services provided. Furthermore, the recruitment strategy, which targeted a specific brothel and three shelter homes in Dhaka, may have introduced selection bias.

## Conclusion

The findings reveal that while most mothers and institutional caregivers have good knowledge and positive attitudes towards their children’s oral health, a significant number of children, especially those living with female sex worker (FSW) mothers, still exhibit poor oral health practices. The study also identified that higher educational attainment among mothers and better oral health knowledge are key predictors of positive oral health practices in children. To address these issues, government-led dental health camps should be organized to meet the oral health needs of this population. Additionally, effective oral health promotion programs are needed to enhance the overall oral health status within this group.

## Electronic supplementary material

Below is the link to the electronic supplementary material.

Supplementary Material 1

Supplementary Material 2

## Data Availability

The datasets used and/or analyzed during the current study are available from the corresponding author on reasonable request.

## Declarations

### Registry and registration no. of the study/trial

The Institutional Review Board /Ethics Review Committee (IRB/ERC) of North South University reviewed and approved this study(2023/OR-NSU/IRB/0204).

## Abbreviations

FSW: Female sex workers
NPO: Not-for-profit organization
NGO: Nongovernmental organization
