# Supplementary material for: Oral health knowledge, attitude and practices of Bangladeshi female sex workers toward their 7-to-17-years old children: a cross-sectional study
Source: BMC Oral Health. 2025 Feb 13;25:233. doi: 10.1186/s12903-025-05535-z (PMC11827219; doi:10.1186/s12903-025-05535-z)
Supplement: Supplementary file 1 — Supplementary Material 1 [file 12903_2025_5535_MOESM1_ESM.docx]

**Table S1: Mother’s/Caregiver’s Response to Oral Health Knowledge on School Children**

| **Items/Questions Regarding Oral Health Knowledge** | **Mother’s/Caregiver’s response** | | | |
| --- | --- | --- | --- | --- |
|  | **Primary School (6-11yearsof age; n=102)** | | **High School (12-17 years of age; n=78)** | |
|  | **Yes** | **No/Don’t know** | **Yes** | **No/Don’t know** |
| **Do you know that teeth are considered an important part of your child’s body?** | 102(100%) | 0(0%) | 76(97.4%) | 2(2.6%) |
| **Do you know that oral health has an impact on general health?** | 95(93.1%) | 7(6.8%) | 64(82.1%) | 14(17.9%) |
| **Do you know that your child should brush his/her teeth twice daily?** | 95(93.1%) | 7(6.8%) | 67(85.9%) | 11(14.1%) |
| **Do you know that, brushing your child’s teeth regularly will prevent oral health problems?** | 99(97.1%) | 3(2.9%) | 73(93.6%) | 5(6.4%) |
| **Do you know that irregular tooth brushing causes toothache?** | 94(92.2%) | 8(7.9%) | 69(88.5%) | 9(11.5%) |
| **Do you know that improper cleaning of your child’s tongue results in bad breath?** | 54(52.9%) | 48(47.1%) | 29(37.2%) | 49(62.8%) |
| **Do you know that frequent consumption of Sweet/Fizzy drinks and/ or sugary snacks can cause oral health problems?** | 82(80.4%) | 20(19.6%) | 64(82.1%) | 14(17.9%) |
| **Do you know that mouth rinsing after having meals, sweets and drinks can prevent oral health problems?** | 58(56.9%) | 44(43.1%) | 57(73.1%) | 21(26.9%) |
| **Do you know that maintaining good oral hygiene prevents tooth decay?** | 89(87.3%) | 13(12.8%) | 71(91%) | 7(9%) |
| **Do you know that carious teeth will affect your child’s teeth appearance?** | 88(86.3%) | 14(13.7%) | 59(75.6%) | 19(24.4%) |
| **Do you know dental treatment can relieve your child’s toothache?** | 90(88.2%) | 12(11.8%) | 69(88.5%) | 9(11.5%) |
| **Do you know that regular dental visits can keep your child’s oral health healthy?** | 91(89.2%) | 11(10.8%) | 70(89.7%) | 8(10.3%) |

**Table S2: Mother’s /Caregiver’s Attitude towards** **Oral Health of School Children**

| **Items/Questions Regarding Oral Health Attitude** | **Mother’s/Caregiver’s response on attitude towards Child’s Oral Health** | | | |
| --- | --- | --- | --- | --- |
|  | **Primary School (6-11yearsof age)** | | **High School (12-17 years of age)** | |
|  | **Agree** | **Disagree** | **Agree** | **Disagree** |
| **Do you think your child should brush his/her teeth twice daily?** | 98(96.1%) | 4(3.9%) | 75(96.2%) | 3(3.8%) |
| **Do you think regular brushing habit will prevent your child from having oral problems?** | 99(97.1%) | 3(2.9%) | 77(98.7%) | 1(1.3%) |
| **Do you think improper cleaning of the tongue results in bad breath?** | 52(51%) | 50(49%) | 22(28.2%) | 56(71.8%) |
| **Do you think poor oral hygiene prevents your child from smiling & laughing with friends?** | 82(80.4%) | 20(19.6%) | 30(38.5%) | 48(61.5%) |
| **Do you think oral problems force your child to miss school?** | 80(78.5%) | 22(21.5%) | 53(67.9%) | 25(32.1%) |
| **Do you think maintaining healthy teeth is an individual responsibility?** | 102(100%) | 0(0%) | 75(96.2%) | 3(3.8%) |
| **Do you think school plays an important role in maintaining oral hygiene?** | 98(96.1%) | 4(3.9%) | 74(94.9%) | 4(5.1%) |
| **Do you think a regular visit to a dentist is necessary?** | 86(84.3%) | 16(15.7%) | 68(87.2%) | 10(12.8%) |
| **Do you think the dentist helps maintain oral health?** | 90(88.2%) | 12(11.8%) | 73(93.6%) | 5(6.4%) |

**Table S3: Mother’s/Caregiver’s Response to Practice Toward Child’s Oral Health**

| **Items/Questions Regarding Oral Health Practices** | **Mother’s/Caregiver’s Response on Practice Toward Child’s Oral Health** | |
| --- | --- | --- |
|  | **Primary School (6-11yearsof age; n= 102)** | **High School (12-17 years of age; n = 78)** |
| **Does your child brush his/her teeth regularly?** | | |
| Yes | 72(70.6%) | 71(91%) |
| No | 30(29.4%) | 7(9.0%) |
| **If not, what reason for not brushing regularly?** | | |
| Lazy | 8(7.8%) | 3(3.8%) |
| Getting up late | 6(5.9%) | 2(2.6%) |
| Don’t like | 16(15.7%) | 2(2.6%) |
| **How often does your child brush his/her teeth regularly?** | | |
| Once | 54(52.9%) | 26(33.3%) |
| Twice or more | 48(47.1%) | 52(66.7%) |
| **Which dentifrice does your child use for brushing his or her teeth every day?** | | |
| Toothpaste | 94(92.2%) | 75(96.2%) |
| Toothpowder | 8(7.8%) | 3(3.8%) |
| **Does he/she brush in front of you?** | | |
| Yes | 64(62.7%) | 50(64.2%) |
| No | 38(37.3%) | 28(35.8%) |
| **How often does your child change your brush?** | | |
| Not Once | 8(7.8%) | 0(0%) |
| Every month | 11(10.8%) | 13(16.6%) |
| Every 2 months | 14(13.7%) | 15(19.2%) |
| Every 3 months | 51(50%) | 44(56.4%) |
| Every 6 months | 15(14.7%) | 6 (7.7%) |
| **Does your child rinse/brush your mouth after meals/sweets/fizzy drinks?** | | |
| Yes | 47(46.1%) | 41(52.6%) |
| No | 55(53.9%) | 37(47.4%) |
| **Does your child clean his/her tongue regularly?** | | |
| Yes | 27(26.9%) | 30(38.5%) |
| No | 75(73.1%) | 48(61.5%) |
