# Supplementary material for: Oral health knowledge, attitude and practices of Bangladeshi female sex workers toward their 7-to-17-years old children: a cross-sectional study
Source: BMC Oral Health. 2025 Feb 13;25:233. doi: 10.1186/s12903-025-05535-z (PMC11827219; doi:10.1186/s12903-025-05535-z)
Supplement: Supplementary file 2 — Supplementary Material 2 [file 12903_2025_5535_MOESM2_ESM.docx]

Sample Size Calculation:

A standard error of 5%, a 95% confidence interval (CI) and a prevalence of 88.5% (37) of mother’s oral health knowledge of regarding their 5 to 9-year-old children in Dhaka city were used for the calculation. Our initial sample size calculation was 156. After adding 20% for the nonresponse rate, our sample size was set to 188 participants. However, after adjusting for missing values and conducting data screening, the final sample size was determined to be 180 subjects.

Z = 1.96 for 95% of the confidence interval

p = proportion of the population who had knowledge about oral health was 88.5% (0.885)

q = 1 − p = 0.8

e = margin of error was at 5% =0.115.

Ƞ = $\frac{\left( \boldsymbol{1}.\boldsymbol{96} \right)^{\boldsymbol{2}}\boldsymbol{x} \boldsymbol{0}.\boldsymbol{885x} (\boldsymbol{1}-0.115)}{(\boldsymbol{0}.\boldsymbol{05})^{2}}$

n = 156
